# Chronic Joint Disease

**Published:** 1884-09

**Authors:** Richard Barwell


### CHRONIC JOINT DISEASE.

In a clinical lecture upon this subject, the author states that in its earlier phases a cure with perfect conservation of movement is possible, and that this termination should always be aimed at and can very often be obtained. The forms of malady referred to generally occur from the first to the twelfth year, and most often attack children of the strumous or scrofulous habit. This is very important. These diseases, arising in strumous children, are rarely of tubercular origin. Struma is a certain habit of body, a certain faulty method of forming and nourishing its tissues, which, thus produced and sustained, are not diseased, but are apt to respond to any slight irritation by sluggish and prolonged inflammations, the immediate products of which have no tendency to higher development, but rather to certain forms of degeneration—suppurative, fatty, caseous and tubercular. In certain parts of the body tubercle appears with great difficulty, and among these the synovial membrane is preeminent. Tubercle does sometimes occur in joints that have long been subject to chronic strumous inflammation, but when so found it is tubercle, not of the synovial membrane, but of old inflammatory products. Again, when tubercle appears in these situations, it is a late production—the terminal not the initial point of a morbid process. These considerations are not mere pedantic hair-splitting, for they underlie the question whether treatment of strumous joint disease is of any great value; for, if the disease were the result of tubercle, the assertion that a cure could often be attained would be false.

In the treatment of these cases the first consideration is to recognize that we have to deal with a malady whose sluggish character depends on a state of bodily mal-nutrition, and one of the essentials toward improving this condition is to abstain from confining the children to one room or ward, and to give them plenty of fresh air and exercise. At the same time, the diseased joint must be kept at rest. The conditions are easily fulfilled when the affected joint is one of the upper extremity, but if it be one of the lower extremity, some difficulties present themselves. Not only must the joint be kept at complete rest, but we must see to it that while moving about no weight is thrown on the joint surface. A large number of splints have been invented for this purpose, and any one can be used that fulfills the conditions in each particular case. On the whole, the one that has given the best satisfaction in our hands is the splint invented by Dr. Dumbrowski, of Dorpat. This is of easy application, and by it the joint is placed in absolute rest, while the remainder of the body is in good exercise.—*Richard Barwell, in The Lancet, August* 2, 1884.

